# Comparative evaluation sealing ability: Bioceramic versus resin-based sealers by micro-CT analysis

**DOI:** 10.6026/973206300220702

**Published:** 2026-02-28

**Authors:** Yelisela Rajiv Kumar, Aboobacker Sidheeque, Siddharth Anand, Bharath Kandanattu, Deepika Khandelwal, Gauri Singh

**Affiliations:** 1 Department of Conservative Dentistry and Endodontics, Government General Hospital, DEIC, RBSK, Eluru, India; 2 Department of Conservative Dentistry and Endodontics, Yenepoya Dental college, Yenepoya University, Mangalore, Karnataka, India; 3 Department of Dentistry, Katihar Medical College and Hospital, Karim Bagh, Sirsa, Katihar, Bihar, India; 4 Department of Pediatric & Preventive Dentistry, Institute of Dental Studies & Technologies, Kadrabad, Modinagar, Uttar Pradesh, India; 5 Department of Conservative Dentistry and Endodontics, Geetanjali Dental and Research Institute, Geetanjali University, Udaipur, India; 6 Department of Prosthodontics and Crown & Bridge, Babu Banarsi Das College of Dental Sciences, lucknow, Uttar Pradesh, India

**Keywords:** Bioceramic sealer, resin-based sealer, micro-CT, sealing ability, root canal obturation

## Abstract

Achieving a hermetic three-dimensional seal of the root canal system remains a primary objective of endodontic therapy, yet comparative evidence on the sealing efficacy of contemporary bioceramic and resin-based sealers remains limited. Therefore, it is interest to compare the sealing ability of three bioceramic sealers (Bio-C Sealer, CeraSeal and EndoSequence BC Sealer HiFlow) with two resin-based sealers (AH Plus and ThermaSeal Plus) using micro-computed tomography. Seventy-five extracted single-rooted human premolars were obturated using single-cone technique for bioceramic sealers and warm vertical compaction for resin-based sealers, followed by three-dimensional assessment of void volume and sealer adaptation. Bioceramic sealers demonstrated significantly lower total void percentages (0.89-1.24%) compared with resin-based sealers (1.78-2.15%) (p < 0.001), with EndoSequence BC Sealer HiFlow showing the best adaptation and lowest void volume. Thus, we show that contemporary bioceramic sealers provide superior sealing ability and canal wall adaptation compared with resin-based sealers, supporting their clinical use for predictable root canal obturation.

## Background:

The basic aim of root canal therapy is total removal of microorganisms in the root canal system and the latter formation of three dimensional hermetic barriers against reinfection and encouraging healing of periapical tissues [1]. Root canal obturation quality has a considerable impact on the final results of treatment by the end of the long term; poor sealing was found to be one of the main causes of endodontic failures via coronal and apical leaks [2]. The modern endodontics practice still struggles to produce a consistent method of creating impermeable seals despite the current improvements in the instrumentation and irrigation methods and the introduction of obturation materials and methods [3]. Root canal sealers play crucial roles in obturation complex such as filler irregularities between gutta-percha and canal walls, penetration of dentinal tubules, lateral canals and anastomoses as well as offering antimicrobial properties and bonding to both dentin and core obturation products [4]. An ideal root canal sealer must have high flow properties, sufficient working time, high dimensional stability, biocompatibility and the capacity to form a fluid-tight seal with clinical conditions [5]. In the past, zinc oxide-eugenol, calcium hydroxide, glass ionomer and resin-based sealer formulations of different successes have been developed to meet these needs [6]. Ah Plus and its derivatives are resin based sealers that have been used in the gold standard to obturate root canals over 20 years because of its favorable physical and long term dimensional stability and clinical performance reportedly [7]. Such sealers exhibit high flow, insolubility and micromechanical retention ability to radicular dentin by the formation of resin tags [8]. Nevertheless, resin based sealers have some weaknesses such as polymerization shrinkage, cytotoxicity during polymerization, may induce allergic responses as well as absence of bioactive features which may enhance periapical healing [9]. With the advent of bioceramic technology, the development of endodontic sealers has taken a new paradigm, as the materials containing calcium silicate chemistry forms have new benefits as compared to traditional ones [10].

Bioceramic sealers are defined as having hydrophilic setting mechanism, biocompatibility, ability to form apatite as well as being antibacterial and having excellent sealing ability due to their expansion during setting [11]. The setting reaction of these materials is completed using moisture in dentinal tubules and the theoretically improved adaptation to canal walls, as well as penetration into anatomical irregularities [12]. Bioceramic sealers such as the iRoot SP and EndoSequence BC Sealer (first generation) showed promising laboratory and clinical results leading to the emergence of more advanced formulations with better characteristics [13]. More recent products, like Bio-C Sealer, CeraSeal and EndoSequence BC Sealer HiFlow are a high-technology bioceramic with altered viscosities, better flow behavior and set times, as a solution to shortcomings of existing products [14]. In these new generation sealers, different ratios of calcium silicates and zirconium oxide as well as proprietary additives are added to produce certain performance attributes [15]. The micro-computed tomography has become the gold standard technique of evaluation of the quality of obturation in the root canals in a non-destructive and three-dimensional manner [16]. This is an imaging modality that allows volumetric measurement of the voids, gaps and sealer distribution throughout the root canal system with resolutions beyond standard radiographic and sectioning methods [17]. The visualization of internal structures and measuring without any physical changes of specimens is of great benefit to the research done on sealer adaptation and formation of voids [18]. Comparative research on bioceramic and resin based sealers has provided inconclusive results in the past with variations in methods of evaluation, obturation processes and sealer agents adding to the conflicting results [19]. Numerous studies have used first-generation bioceramic sealers or have used two-dimensional assessment processes which are unlikely to be sensitive to three dimensional sealing properties [20]. Moreover, the literature does not include direct comparisons between several modern bioceramic formulations [21]. Therefore, it is of interest to evaluate the sealing ability of contemporary bioceramic sealers in comparison with resin-based sealers using micro-computed tomography-based volumetric analysis.

## Materials and Methods:

## Design of the study and sampling:

This experimental research work was carried out *in vitro* with the approval of the institutional ethics committee. G + power software (version 3.1.9.7) was used to calculate the sample size based on effect size of 0.45 obtained in the preliminary data, alpha error of 0.05 and statistical power of 0.85, which showed that a minimum number of 13 specimens was required per group. In order to deal with the possibility of loss of the specimens, 15 teeth were chosen per group which were 75 specimens. Premolars of the human mandibular and maxillary jaws extracted due to orthodontic or periodontic reasons were gathered with patient informed consent. Inclusion criteria included single-rooted premolars with single canals that were radiographically verified, fully formed apices, no caries or restorations in the region of the cementoenamel junction, no visible cracks or fractures under magnification, patent canals negotiable to the apical foramen, the root curvature was less than 25°C according to the Schneider method and the length of the root was 13-17mm. The exclusion criteria were the presence of teeth with multicellular canals, prior endodontic therapy, anatomical abnormalities and internal or external resorption. Storage of teeth was done in thymol solution of 0.1% at 4°C no more than three months after extraction. Before experimental procedures, soft tissue remnants and calculus deposits were removed using ultrasonic scalers on the specimens and the observation performed under dental operating microscope at 10x magnification to ensure that there were no cracks.

## Specimen preparation:

Standardized access cavities were made with a high-speed handpiece using diamond burs and water cooling. The length of a work was determined by placing a size 10 K-file until the tip appeared at the apical foramen, then dividing the result by 0.5mm. The canal patency was verified and recorded. The instrumentation of root canals was done in the ProTaper Gold rotary system (Dentsply Sirona, Charlotte, NC, USA) as per the manufacturer instructions. Coronal flaring took place with shaping files S1 and S2 and finishing files F1, F2 and F3 were brought to the desired working length, creating a standard size of preparation of 30/.09 taper at apical end. The instrumentation was done with an endodontic motor (X-Smart Plus, Dentsply Sirona) with a torque of 300 rpm and 2.5 Ncm. Irrigation regimen included 3mL of 2.5 percent sodium hypochlorite between instrument changes which was administered using 30-gauge side-vented needles placed 2mm below working length. End irrigation was 5mL of 17% EDTA 1 minute and 5 mL of 2.5 percent sodium hypochlorite. Sterile paper points were used to dry canals.

## Group allocation and obturation:

Five experimental groups (n=15 each) were formed with prepared specimens assigned randomly using the computers:

[1] Group 1 - Bio-C Sealer (Angelus, Londrina, Brazil): This is a calcium silicate-based bioceramic sealer that was used with the matched-taper single-cone technique and ProTaper F3 gutta-percha points.

[2] Group 2 - CeraSeal (Meta Biomed, Cheongju, Korea): Premixed calcium silicate bioceramic sealer with matching gutta-percha points and matched taper points delivered by single-cone with matched gutta-percha point in matching taper technique.

[3] Group 3 - EndoSequence BC Sealer HiFlow (Brasseler USA, Savannah, GA, USA): BIOCeramic sealer with a high flow and used in warm obturation techniques, which is applied with matched-taper single-cone technique.

[4] Group 4 - AH Plus (Dentsply Sirona, Konstanz, Germany): Epoxy resins-based sealer fitted with warm vertical compaction technique with System B heat source and Obtura III Max backfill system.

[5] Group 5 - ThermaSeal Plus (Dentsply Sirona, York, PA, USA): A resin-based sealer with altered composition, which is applied with the help of warm vertical compaction.

With bioceramic sealer groups, sealers were placed onto canal walls with the tips or lentulo spirals that were supplied by the manufacturers at 300 rpm. One, matched-taper gutta-percha cone with a sealer layer was placed to working length and excess of material was trimmed off at the level of the orifice by a heated plugger. With resin based sealer groups, sealers were prepared as per the instructions by the manufacturers and applied on the canal walls. A master cone of the working length which has a tug-back sensation was chosen. Warm vertical compaction was carried out on the continuous wave method whereby the heat carrier was at 200°C and then the backfill was carried out with thermoplasticized gutta-percha in 4mm layers. Obturation procedures were done by one experienced operator. Cavities were sealed with Cavit (3M ESPE, St. Paul, MN, USA) temporarily and stored at 37°C and 100 percent humidity, to ensure full sealer cure. Micro-CT Scanning Protocol Micro-CT scanning Micro-CT scanning was conducted in a high-resolution scanner (SkyScan 1275, Bruker, Kontich, Belgium) with the following acquisition parameters as follows: source voltage 100kV, source current 100 u A, pixel size 8 u M, rotation step 0.3, 360 rotation, aluminum/copper filter with frame averaging of 4. Specimens were attached in custom acrylic holders so that they could be positioned in a consistent way. Image reconstruction was done in NRecon software (version 1.7.4.6, Bruker) with standard parameters of beam hardening correction of 40, ring artifact reduction of 6 and smoothing of 1. The reconstructions data included about 1,800-2,200 axial slices of a specimen.

## Three-dimensional analysis:

Two blinded examiners (to group allocation) used CTAn software (version 1.20.3.0, Bruker) to apply volumetric analysis. The area of focus was considered the obtained root canal space between the canal orifice and apical terminus. The global thresholding was used to make segmentations of gutta-percha, sealer and voids as well as the dentin in terms of their different radiodensities. The threshold values were established using the histogram analysis and confirmed with pilot specimens whose compositions were known.

## Parameters that were measured were:

[1] Total void volume (mm 3): Volume of any voids and spaces in the obturation.

[2] Percent void (%)/: Ratio of the void volume to the volume of canal space.

[3] Sealer volume (mm 3): Sealer volume in the obturation.

[4] Sealer percentage (percent): Fraction of sealer to the volume of obturation.

[5] Gutta-percha volume (mm 3): Core Material Gutta-percha volume.

[6] Interfacial gap volume (mm 3): Voids those are present at the interfaces of sealer and dentin.

Regional analysis involved three parts of each root namely coronal third, middle third and apical third. Each region was measured in terms of void distribution.

## Quality assessment of adaptation:

Semi-quantitative system of adaptation scoring was done on standardized axial sections at 3mm, 6mm and 9mm in relation to apical terminus:

[1] Score 0: No visible points of discontinuity.

[2] Score 1: Minor gaps (Less than 25 percent circumference)

[3] Score 2: Moderate gaps (25-50 percent circumference)

[4] Score 3: Large gaps (more than half circumference)

## Statistical analysis:

The statistical analysis was done using SPSS (version 27.0, IBM Corp., Armonk, NY, USA). Shapiro-Wilk tests were used to measure normal distribution of data. Mean standard deviation was used to present continuous variables. One-way ANOVA with a post-hoc test (normally distributed data) was used to compare groups between-groups. Two-way ANOVA was used to test the distribution of regional voids. The Kruskal-Wallis test with Dunn post-hoc analysis was used to compare the scores of adaptation. The inter-examiner reliability was determined by means of intraclass correlation coefficients. The level of statistical significance was set at p<0.05.

## Results:

All 75 specimens completed the study protocol without exclusions. Inter-examiner reliability demonstrated excellent agreement with intraclass correlation coefficients ranging from 0.91 to 0.96 for all volumetric measurements. Bioceramic sealer groups exhibited significantly lower void volumes compared to resin-based sealer groups. Mean void percentage ranged from 0.89% to 1.24% for bioceramic sealers compared to 1.78% to 2.15% for resin-based sealers. EndoSequence BC Sealer HiFlow demonstrated the lowest void percentage (0.89±0.31%), while AH Plus showed the highest (2.15±0.72%). One-way ANOVA revealed significant differences among groups (F=28.45, p<0.001). Post-hoc analysis confirmed that all bioceramic sealers demonstrated significantly lower void percentages compared to both resin-based sealers (p<0.001 for all comparisons). Among bioceramic sealers, EndoSequence BC Sealer HiFlow showed significantly lower void volume than Bio-C Sealer (p=0.024), while CeraSeal demonstrated intermediate values not significantly different from either (p>0.05). Detailed void analysis results are presented in Table 1. Bioceramic sealer groups demonstrated higher sealer percentages within the obturation compared to resin-based groups, consistent with the single-cone technique employed. Mean sealer percentage ranged from 28.4% to 31.2% for bioceramic groups versus 18.6% to 21.3% for resin-based groups. Gutta-percha volume was correspondingly higher in resin-based sealer groups (78.5-81.4%) compared to bioceramic groups (68.8-71.6%), reflecting the different obturation techniques. Canal filling percentage (gutta-percha plus sealer, excluding voids) was significantly higher in bioceramic groups (98.8-99.1%) compared to resin-based groups (97.9-98.2%). Complete distribution data are shown in Table 2. Two-way ANOVA revealed significant effects of both sealer type (p<0.001) and root region (p<0.001) on void percentage, with significant interaction (p=0.012). All groups demonstrated highest void percentages in the apical third compared to coronal and middle thirds. Bioceramic sealers showed more uniform void distribution across root thirds compared to resin-based sealers, which exhibited pronounced increases in apical void percentages. The apical-to-coronal void ratio was 1.42 for bioceramic groups versus 2.18 for resin-based groups. Adaptation quality scores demonstrated significant differences among groups (Kruskal-Wallis H=42.87, p<0.001). EndoSequence BC Sealer HiFlow exhibited the highest proportion of score 0 (complete adaptation) assessments (82.2%), while AH Plus showed the lowest (51.1%). Regional distribution and adaptation data are presented in Table 3. Pearson correlation analysis revealed significant negative correlations between sealer percentage and void percentage (r=-0.52, p<0.001), suggesting that higher sealer volumes were associated with reduced void formation. Positive correlation existed between void percentage and adaptation scores (r=0.68, p<0.001), confirming the relationship between volumetric voids and circumferential gaps.

## Discussion:

This research study has shown that the new generation bioceramic sealers have better sealing capacity than the existing resin-based sealers when measured by using the micro-CT volumetric analysis. The data confirms the null hypothesis and adds to the accumulating body of evidence that bioceramic materials are better options to use to obturite root canals [22]. The much lower levels of void percentages in bioceramic sealer samples (0.89-1.24) and resin-based sealer samples (1.78-2.15) are in agreement with earlier studies that reported superior adaptation of calcium silicate-based sealer materials to radicular dentin [23]. This high sealing effect can be seen to be as a result of the hydrophilic setting mechanism of bioceramic sealers that make use of moisture contained in dentinal tubules to accomplish the setting reaction and also enhance close contact with canal walls [24]. The minor growth of the bioceramic sealers as they set is another process that leads to improved formation of seals. There has been a record of expansion of up to 0.02 to 0.06 with calcium silicate sealers in comparison to shrinkage of resin-based sealers of 0.5 to 1.5 percent on polymerization [23]. This dimensional behavior has a direct effect on the interfacial gap formation, where shrinkage forms potential leakage paths of bacteria whereas expansion preserves or enhances adaptation [26]. EndoSequence BC Sealer HiFlow reflected the least void percentage of all sealers tested and this could be explained by its explicitly increased flow properties that were aimed at the better penetration into the anatomical irregularities and lateral canals [27]. The Hi Flow formulation contains adjustments of the particle size distribution and carrier medium viscosity that maximizes the flow behavior without altering the appropriate handling properties [28]. The described properties can help fill the canal space more fully during obturation. The increased sealer percentages in the bioceramic groups are a result of the single-cone obturation method used which naturally results in higher volumes of sealer than the warm vertical compaction [29]. Although thought to be disadvantageous the dissolution of sealer, modern bioceramic sealers exhibit insignificant dissolution under full setting, possibly overcoming this factor [30]. Moreover, calcium silicate sealers have bioactive properties that might be beneficial in it, such as the formation of hydroxyapatite at the sealer-dentin interface, which could be of further benefit other than merely space-filling [31]. The distribution of the regional voids analysis showed that the percentages of the voids are more in all groups at the apical third as it is an anatomically complex region and less accessible [32]. Nevertheless, bioceramic sealers showed better uniformity of the void distribution with lower ratio of apical and coronal which indicates better flow properties to allow appropriate apical adaptation.

The strong resin-based sealers induced changes in the number of apical voids can be associated with the technical difficulties in the total compaction of warm gutta-percha in small apical shapes [33]. Warm vertical compaction technique used in resin based sealers although a gold standard technique in theory can cause void formation in a number of ways [34]. Sealer can be sped up by using heat which may hamper flow before full adaptation is attained. Also, the compaction forces can push in place partially set sealer, resulting in interfacial gaps [35]. These technical considerations indicate the significance of placing sealer-technique compatibility in clinical decisions. The quality scoring of adaptation coincided with the volumetric results showing bioceramic sealers fully adjusted the circumferential sections of 75.6-82.2 against 51.1-60.0 of resin-based sealers [36]. This qualitative measure offers clinically useful data on the possible leakage routes, which cannot be fully measured by use of volumetric methods [37]. This research has significant clinical implications with the superior performance of bioceramic sealers. The disadvantage of void formation leads to a reduction of the potential of bacteria leakage which has been associated with enhanced long-term treatment outcomes [38]. Moreover, calcium silicate sealers have the potential to enhance periapical healing as their biocompatibility, as well as bioactivity is not just a result of physical sealing activities, but additional anti-inflammatory responses and mineralization effects that calcium silicate sealers may provoke [39]. Negative correlation between sealer percentage and void percentage in this study is worth taking special meanings. Although an increase in volumes of the sealer was correlated with a decrease in the number of voids in the obturation, excess sealer can be unwanted because of the possible mechanical flaws [40]. The optimum sealer thickness is a compromise between sufficient coverage to form a seal and reduction of material bulk, which may be degraded or fail [41]. There are some limitations that this study has and which it is important to mention when generalizing the findings into clinical practice. The *in vitro* design excludes variables found in clinical conditions such as saliva contamination, failure to achieve hemostasis and patient factors having effect on obturation quality [42]. Using single- rooted premolars with a fairly simple anatomy could be not reflective of the difficulties that arise in complex multi-rooted teeth with anatomical variations. The various obturation methods used with bioceramic and resin-based sealers though they may be representative of current clinical practice incorporate a confounding variable that does not allow the direct evaluation of the performance of sealers without the influence of the methods used [43]. Further studies comparing sealers applied with the same obturation protocols can give other information on material-specific effects on sealing ability.

## Conclusion:

The new generation bioceramic sealers exhibited much better sealing ability than the resin based sealers in the determination of micro-CT volumetric analysis. EndoSequence BC Sealer HiFlow showed the minimum values of void formation and the highest value of adaptation quality and CeraSeal and Bio-C Sealer showed the intermediate values of the same, whereas AH Plus showed the highest values of the percentage of voids among all sealers tested. Bioceramic sealers also exhibited a more evenly distributed void in root thirds and had complete circumferential adaptation in a substantially greater percentage of sections studied.

## Figures and Tables

**Table 1:** Volumetric void analysis comparison among sealer groups

**Parameter**	**Bio-C Sealer (n=15)**	**CeraSeal (n=15)**	**BC Sealer HiFlow (n=15)**	**AH Plus (n=15)**	**ThermaSeal Plus (n=15)**	**p-value**
Total void volume (mm^3^)	0.156±0.048	0.142±0.039	0.118±0.041	0.287±0.095	0.237±0.078	<0.001*
Void percentage (%)	1.24±0.38^a^	1.08±0.32^ab^	0.89±0.31^b^	2.15±0.72^c^	1.78±0.59^c^	<0.001*
Interfacial gap volume (mm^3^)	0.089±0.028	0.078±0.024	0.062±0.022	0.168±0.056	0.142±0.048	<0.001*
Interfacial gap percentage (%)	0.71±0.22^a^	0.59±0.19^a^	0.47±0.17^b^	1.26±0.42^c^	1.07±0.36^c^	<0.001*
Maximum void size (mm^3^)	0.024±0.009	0.021±0.008	0.018±0.007	0.045±0.018	0.038±0.015	<0.001*
*Values presented as mean ±
standard deviation; Different superscript
letters indicate statistically significant
differences (p<0.05);
Statistically significant

**Table 2:** Sealer and gutta-percha distribution analysis

**Parameter**	**Bio-C Sealer (n=15)**	**CeraSeal (n=15)**	**BC Sealer HiFlow (n=15)**	**AH Plus (n=15)**	**ThermaSeal Plus (n=15)**	**p-value**
Total canal volume (mm^3^)	12.58±1.87	13.15±2.04	13.27±1.95	13.34±2.12	13.31±1.98	0.782
Gutta-percha volume (mm^3^)	8.78±1.34	9.04±1.42	9.18±1.38	10.47±1.68	10.84±1.72	<0.001*
Gutta-percha percentage (%)	69.8±4.2^a^	68.8±4.5^a^	69.2±4.1^a^	78.5±5.1^b^	81.4±4.8^b^	<0.001*
Sealer volume (mm^3^)	3.64±0.58	3.97±0.62	3.99±0.59	2.60±0.48	2.24±0.42	<0.001*
Sealer percentage (%)	28.9±3.8^a^	30.2±4.1^a^	30.1±3.9^a^	19.5±3.2^b^	16.8±2.9^b^	<0.001*
Canal filling percentage (%)	98.76±0.38^a^	98.92±0.32^a^	99.11±0.31^a^	97.85±0.72^b^	98.22±0.59^b^	<0.001*
*Values presented as mean ±
standard deviation;
Different superscript letters indicate
statistically significant differences
(p<0.05); Statistically significant

**Table 3:** Regional void distribution and adaptation quality scores

**Parameter**	**Bio-C Sealer (n=15)**	**CeraSeal (n=15)**	**BC Sealer HiFlow (n=15)**	**AH Plus (n=15)**	**ThermaSeal Plus (n=15)**	**p-value**
**Regional Void Percentage (%)**						
Coronal third	0.87±0.28	0.76±0.24	0.64±0.21	1.42±0.48	1.18±0.39	<0.001*
Middle third	1.12±0.35	0.98±0.31	0.82±0.27	1.89±0.62	1.56±0.51	<0.001*
Apical third	1.73±0.52	1.51±0.45	1.21±0.38	3.14±0.98	2.61±0.82	<0.001*
Apical/Coronal ratio	1.99±0.42	1.99±0.38	1.89±0.35	2.21±0.58	2.21±0.52	0.089
**Adaptation Scores, n (%)**						
Score 0 (Complete)	34 (75.6%)	35 (77.8%)	37 (82.2%)	23 (51.1%)	27 (60.0%)	<0.001*
Score 1 (Minor gaps)	8 (17.8%)	7 (15.6%)	6 (13.3%)	12 (26.7%)	11 (24.4%)	
Score 2 (Moderate gaps)	2 (4.4%)	2 (4.4%)	2 (4.4%)	7 (15.6%)	5 (11.1%)	
Score 3 (Extensive gaps)	1 (2.2%)	1 (2.2%)	0 (0%)	3 (6.7%)	2 (4.4%)	
Median adaptation score	0	0	0	0	0	<0.001*
*Values presented as mean ±
standard deviation or n (%);
Statistically significant (p<0.05)
